# Selected papers from the 14th Annual Bio-Ontologies Special Interest Group Meeting

**DOI:** 10.1186/2041-1480-3-S1-I1

**Published:** 2012-04-24

**Authors:** Larisa N Soldatova, Susanna-Assunta Sansone, Michel Dumontier, Nigam H Shah

**Affiliations:** 1 Aberystwyth University, Wales, UK; 2 University of Oxford, Oxford e-Research Centre, UK; 3 Carleton University, Ottawa, Canada; 4 Stanford University, CA, USA

## Abstract

Over the 14 years, the Bio-Ontologies SIG at ISMB has provided a forum for discussion of the latest and most innovative research in the bio-ontologies development, its applications to biomedicine and more generally the organisation, presentation and dissemination of knowledge in biomedicine and the life sciences. The seven papers selected for this supplement span a wide range of topics including: web-based querying over multiple ontologies, integration of data from wikis, innovative methods of annotating and mining electronic health records, advances in annotating web documents and biomedical literature, quality control of ontology alignments, and the ontology support for predictive models about toxicity and open access to the toxicity data.

## Summary of selected papers

In 2011, the SIG received 29 paper submissions, 5 flash updates and 4 poster abstracts. 18 papers and 11 flash updates (some papers were converted to flush updates) were selected for presentation at the meeting, out of which 7 appear in this supplement.

The seven papers selected for this supplement are extended versions of the original papers presented at the 2011 SIG. The papers include research on web-based querying over multiple ontologies [3], integration of data from wikis into a meta semantic wiki [6], advances in the annotation of electronic health records [5], web documents [1] and biomedical literature [2], quality control of ontology alignments [4], and the ontology based support for the toxicity predictive models and access to the toxicity data [7].

Paolo Ciccarese et al in the paper titled “DOMEO: a web-based tool for semantic annotation of online documents” describe work to date on an advancement in scientific document automated annotation and collaborative curation using integrated ontologies and frameworks in bioscience. The authors report on a user-friendly web based interface Domeo for the implementation of annotations of existing web resources.

Domeo is an extensible web application enabling users to visually and efficiently create and share ontology-based stand-off annotation on HTML or XML document targets. The Domeo annotation tool supports manual, fully automated, and semi-automated annotation with complete provenance records, as well as personal or community annotation with access authorization and control.

Domeo has been deployed in beta release as part of the NIH Neuroscience Information Framework (http://www.neuinfo.org). The Domeo annotation tool is available at http://annotationframework.org/.

The paper titled “A maximum entropy approach for accurate document annotation” by George Tsatsaronis et al addresses the critical task of document tagging for annotation of biomedical literature against ontologies of medical concern such as anatomy, disease and phycology. The authors present an automated and robust method, based on maximum-entropy approach, for annotating biomedical literature documents with terms from Medical Subject Headings (MeSH). The suggested approach has increased F-measure results when tested against title, journal and abstract text even using a small number of documents. The work demonstrates the potential benefit of using ontologies to address the problem of information retrieval/extraction given the exponential growth of biomedical literature.

Simon Jupp et al in the paper titled “Exploring Gene Ontology annotations with OWL” demonstrate that the controlled vocabulary annotations of gene products using GO terms can be engineered to represent logical property restrictions on classes and individuals in the OWL language such that it becomes possible to execute rich, logical queries over genes utilizing both the semantics of the gene annotations, as well as the semantics of the GO itself. To do multi-perspective rich querying, the authors created the GOAL (Logical Gene Ontology) that combines the Gene ontology, Human Disease Ontology, and the Mammalian Phenotype Ontology, together with classes for mouse gene products. Defined classes were used to query these protein classes through automated reasoning. This was presented through a Web interface that allows rich queries and displays results. The GOAL ontology is available at http://owl.cs.manchester.ac.uk/goal.

The paper “Towards valid and reusable reference alignments – ten basic quality checks for reference alignments and their application to three different reference data sets” by Elena Beisswanger and Udo Hahn outlines work in the area of ontology alignment and highlights different checks that should be completed to assess whether alignment is valid.

The basic quality checks maybe of help to editors and curators evaluating the alignment of ontologies, focusing on the reliability and reusability aspects.

The checks are applied to the anatomy reference alignment datasets (the anatomy branch of the NCI Thesaurus and the Mouse adult gross anatomy ontology), also used in the yearly Ontology Alignment Evaluation Initiative (OAEI); to the alignments of Linked Open Data schemes; and for assessing upper ontology-based alignment approaches. The results reveal incorrect and missing correspondences, classes, and missing or invalid relations between classes in the input ontologies. The basic checks contribute to the quality of alignments. This is an area of growing importance.

In “Annotation analysis for testing drug safety signals” by Paea LePendu et al describe a novel approach for leveraging resources for biomedical computing, such as the NCBO Annotator tool and public biomedical ontologies for mining electronic health records and to test drug safety signals. The authors analyzed over 9 million unstructured clinical notes to compute the risk of having a myocardial infarction after taking Vioxx prescribed for rheumatoid arthritis. Their analysis reveals significant elevated risk for myocardial infarction in rheumatoid arthritis patients taking Vioxx as early as 2003 (2 years before the actual recall of the drug). The results demonstrate the utility of applying ontology based annotation analysis for detecting the relationships between drugs and health conditions in general. The results also demonstrate the feasibility of text-mining based approach for early detection of drug associated adverse events by exploring a corpus of electronic health records.

In the paper titled “Linking genes to diseases with a SNPedia-Gene Wiki mashup”, Benjamin Good et al present a semantic meta-wiki called the Gene Wiki+ that automatically integrates data from Gene Wiki and SNPedia. The Gene Wiki provides continuously updated review articles for human genes, including descriptions of the role the gene may play in disease, and SNPedia provides textual information about links between variations in human genes and human phenotypes. The Gene Wiki+ exposes substantially more evidence of links between genes and diseases than either resource contains independently. Currently, it captures >8,000 distinct gene-disease relationships. The authors created an open source program SyncBot that continuously monitors the source wikis for changes and ensures that the Gene Wiki+ stays up-to-date. The content of the Gene Wiki+ is exported as RDF. The Gene Wiki+ is available at http://genewikiplus.org.

Olga Tcheremenskaia et al in the paper titled “OpenTox predictive toxicology framework: toxicological ontology and semantic media wiki-based OpenToxipedia” report on a suite of ontologies developed for the OpenTox project (http://www.opentox.org). The OpenTox framework aims at providing access to toxicity data, predictive models, and validation procedures. Interoperability of resources is based on the OpenTox ontologies describing predictive algorithms, models, and toxicity data. These ontologies have been developed following the OBO Foundry principles. The authors also report on wiki for the OpenTox predictive toxicology framework. The described project is of major importance and likely to lead to many results in toxicology. The OpenTox ontologies are available at http://www.opentox.org/dev/ontology.
